# Characterizing short germline-specific promoters with a range of expression levels in *C. elegans*

**DOI:** 10.17912/micropub.biology.000843

**Published:** 2023-07-10

**Authors:** Mohammed D. Aljohani, Sonia El Mouridi, Christian Frøkjær-Jensen

**Affiliations:** 1 King Abdullah University of Science and Technology (KAUST), Biological and Environmental Science and Engineering Division (BESE), KAUST Environmental Epigenetics Program (KEEP), Thuwal, 23955-6900, Saudi Arabia.

## Abstract

A core tenet of synthetic biology is that well-characterized regulatory elements are essential for engineering biological systems. Here, we characterize the specificity and expression levels of 18 short (254 to 880 bp) candidate germline promoters using a single-copy *gfp* reporter assay in *C. elegans* . Six promoters resulted in ubiquitous expression, three did not drive detectable expression, and nine were germline-specific. Several promoters drove stronger germline expression than the commonly-used *mex-5* promoter. The promoters range across expression levels and facilitate, for example, low expression of toxic transgenes or high expression of gene editing enzymes, and their compactness facilitates gene synthesis.

**Figure 1. Functional Characterization of Germline-Specific Promoters in C. elegans:**
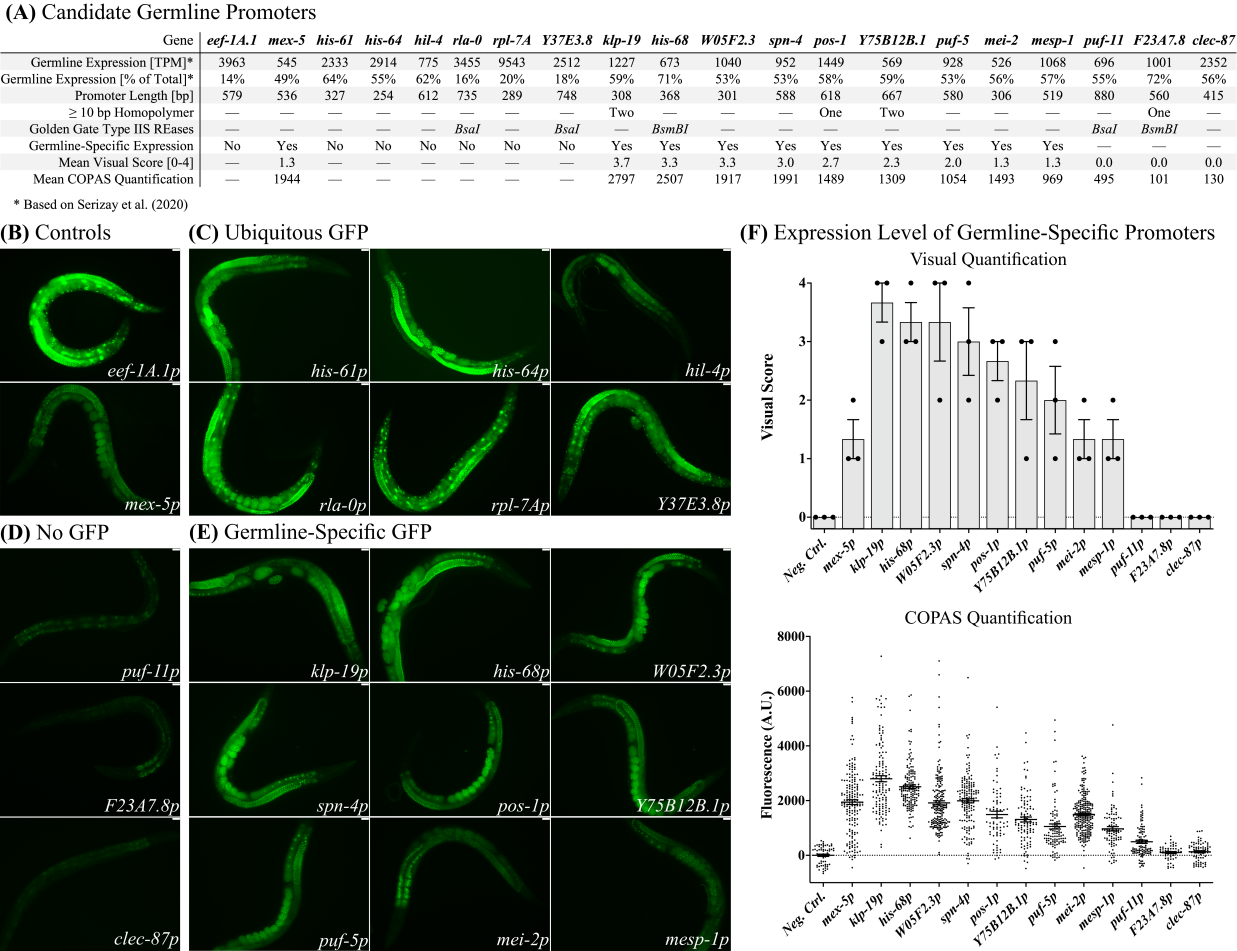
**(A)** Properties of selected candidate promoters and summary of results. RNA-seq data from Serizay et al. (2020) is reported in Transcript Per Million (TPM). We calculated the relative germline expression as a fraction of expression in all major tissues (germline, neurons, intestines, hypodermis, and muscles). We selected promoter regions from the start codon of the candidate gene to the start or stop codon of the adjacent protein-coding gene. Homopolymer stretches of 10 bp or more and *BsaI* type IIS restriction sites in the selected promoter regions were modified by the introduction of base substitutions to facilitate synthesis and Golden Gate assembly. ( **B)** Expression patterns of the ubiquitous *eef-1A.1 p* (alternative name *eft-3p* ) and germline *mex-5 p* controls. **(C-E)** We classified germline-specific candidate promoters by careful visual inspection for somatic expression on a fluorescence microscope at high magnification (40x, oil objective). Due to very high GFP expression in the germline, somatic expression is not easily visible in all images. **(C)** GFP expression patterns of promoters that showed both somatic and germline fluorescence: *his-61 p* , *his-64 p* , *hil-4 p* , *rla-0 p* , *rpl- 7Ap* , and *Y37E3.8 p* . **(D)** Promoters with no detectable GFP expression: *puf-11 p* , *F23A7.8 p,* and *clec-87 p* . **(E)** Promoters with germline-specific expression: *klp-19 p* , *his-68 p* , *W05F2.3 p* , *spn-4 p* , *pos-1 p* , *Y75B12B.1 p* , *puf-5 p* , *mei-2 p* , and *mesp-1 p* . Images were taken using a 20x air objective. Scale bars = 20 μm. **(F)** Top: Visual quantification of germline-specific GFP expression by scoring transgenic animals (blinded to genotype) from 0 (no expression) to 4 (high expression) on a fluorescence dissection microscope. Neg. Ctrl. refers to non-transgenic N2 animals. Bars indicate the mean, and error bars indicate the SEM. Bottom: Expression measurements using COPAS flow cytometry (Neg. Ctrl. (N = 64), *mex-5 p* (N = 179), *klp-19 p* (N = 134), *his-68 p* (N = 156), *W05F2.3 p* (N = 195), *spn-4 p* (N = 160), *pos-1 p* (N = 75), *mei-2 p* (N = 238), *Y75B12B.1 p* (N = 103), *puf-5 p* (N = 104), *mesp-1 p* (N = 91), *puf-11 p* (N = 109), *F23A7.8 p* (N = 54), *clec-87 p* (N = 75)). Values plotted are relative to the mean of Neg. Ctrl. (non-transgenic N2 animals). Lines indicate the mean, and error bars indicate the SEM.

## Description

The precise manipulation of biological systems requires a versatile synthetic biology toolkit of regulatory elements. Libraries of standardized genetic “parts” have permitted control over complex metabolic pathways to produce valuable chemicals or introduce novel traits in various biological systems, including bacteria, yeast, and plants (Choi et al., 2019) . Germline promoters are particularly useful for heritable genome editing, as well as the study and manipulation of germline processes. Precise control over transgene expression levels is advantageous, as protein function and toxicity are generally dosage-dependent. For example, in *C. elegans,* overexpression of the microtubule force regulator GPR-1 by codon adaptation was able to change Mendelian inheritance by forcing premature cell division in the early embryo (Redemann et al., 2011; Besseling & Bringmann, 2016) . As an alternative approach to modulate transgene expression, Artiles et al. (2019) generated random *gpr-1* :: *gfp* insertions and relied on position effect variegation to isolate viable lines with "Goldilocks" expression: stable and exactly enough expression to generate a high frequency of non-Mendelian inheritance but no obvious toxicity. Moreover, while two germline promoters ( *mex-5 p* and *pie-1 p* ) are frequently used in *C. elegans* , ubiquitous promoters with high germline expression ( *eef-1A.1 p* (prior nomenclature *eft-3p* ), *smu-1 p* , and *smu-2 p* ) are often used for efficient genome editing (Frøkjær-Jensen et al., 2012; Aljohani et al., 2020). These examples highlight the importance of controlling transgene expression to engineer biological systems and generate desired outcomes. We reasoned that advances in tissue-specific sequencing provide new opportunities for the rational identification of regulatory components with particular characteristics. Here, we characterize short putative germline promoters across a range of expression levels in *C. elegans* .

We identified candidate promoters using tissue-specific RNA-seq data from Serizay et al. (2020) and selected short promoters (less than 1 kb) from genes with high absolute and relative germline expression ( **Figure 1A** ). To facilitate synthesis and cloning, we modified homopolymers of 10 bp or more and *BsaI* recognition sites using single nucleotide substitutions. We cloned candidates and controls (ubiquitous *eef-1A.1 p* and germline *mex-5 p* ) into vectors containing a codon-optimized *gfp* with nuclear localization signals and a germline permissive 3’ UTR ( *tbb-2* ) using Golden Gate Assembly (Merritt et al., 2008; Engler et al., 2009; Fielmich et al., 2018) . We tested the expression of each transgene from single-copy insertions into a germline-permissive safe-harbor landing site on chromosome II (8.24 Mb) using MosTI (El Mouridi et al., 2022) .

We verified that the constructs and insertion strategy produce ubiquitous and germline *gfp* expression using the standard *eef-1A.1 p* and *mex-5 p* promoters ( **Figure 1B** ). We then assessed the tissue-specificity and expression patterns of each candidate promoter using fluorescence microscopy. We observed GFP expression in somatic cells in six candidates ( *his-61 p* , *his-64 p* , *hil-4 p, rla-0 p* , *rpl-7A p* , and *Y37E3.8 p)* in addition to the germline ( **Figure 1C** ). Expression in the soma from *his-61 p* , *his-64 p* , and *hil-4 p* was limited compared to the broad expression found using *rla-0 p* , *rpl-7A p* , and *Y37E3.8 p* . The specificity of these promoters reflects the relative germline expression measurements from RNA-seq data ( **Figure 1A** ). Although multiple candidates are expressed in the soma and the germline, promoters such as *his-61 p* (327 bp) and *his-64 p* (254 bp) provide shorter alternatives to *eef-1A.1 p* (579 bp).

We observed no detectable GFP expression from *puf-11 p* , *F23A7.8 p,* and *clec-87 p* reporter constructs ( **Figure 1D** ), despite the high germline RNA expression for *clec-87* (2,352 TPM) and the highest fractional germline expression for *F23A7.8 p* (72%) ( **Figure 1A** ). The discrepancy with RNA-seq data could be due to regulation in the native genomic context outside the selected promoter region. ATAC-seq data show potential germline enhancers for *clec-87* and *puf-11* near their 3' UTR (Serizay et al., 2020) , suggesting possible distal regulation of germline expression. However, no clear open chromatin peaks are reported at the *F23A7.8* locus. *F23A7.8* is the only candidate gene selected from chromosome X, which is mostly silenced in the germline (Kelly et al., 2002) . These results highlight the need to experimentally test putative regulatory elements, and these promoters could potentially serve as minimal promoters to screen for distal cis-regulatory elements.

Nine promoters ( *klp-19 p* , *his-68 p* , *W05F2.3 p* , *spn-4 p* , *pos-1 p* , *Y75B12B.1 p* , *puf-5 p* , *mei-2 p* , and *mesp-1 p* ) produced GFP expression that was specific to the germline ( **Figure 1E** ). We quantified GFP expression levels using two assays: a blinded visual screen under a fluorescence dissection microscope and using a COPAS flow cytometer ( **Figure 1F** ). The relative expression levels generally correlated across the two quantification methods, with the exception of *mei-2 p* and *mex-5 p* . The quantification revealed a range of expression levels in the germline. Notably, *klp-19 p* and *his-68 p* consistently produced GFP that is brighter than *mex-5 p* . These promoters provide promising options to enhance gene editing efficiency and limit somatic background with high and specific expression levels in the germline. *puf-5 p* , *pos-1 p* , and *Y75B12B.1 p* offer medium expression levels, and *mei-2 p* and *mesp-1 p* offer relatively low expression levels. These promoters are well-suited for experiments that require modest protein levels or to reduce transgene toxicity in the germline. The relatively short length of these promoters makes them practical for gene synthesis. Therefore, we have incorporated the sequences of *his-64 p* for ubiquitous and *klp-19 p* (high), *Y75B12B.1 p* (medium), and *mesp-1 p* (low) for germline expression in an online application (https://www.wormbuilder.org/transgenebuilder/) (Vargas-Velazquez, El Mouridi, Alkhaldi, and Frøkjær-Jensen, manuscript in preparation) for convenient transgene design and synthesis.

In summary, we characterized a set of germline-specific promoters that allow control over a range of expression levels in the germline. Our findings highlight the need for functional validation of RNA-seq and ATAC-seq data to annotate promoters. The newly characterized regulatory elements expand the growing *C. elegans* synthetic biology toolbox with short promoters that fine-tune germline expression and provide the means to regulate biological pathways with precision, improve genome editing specificity and efficiency, and mitigate potential transgene toxicity.

## Methods

**Strains**

We maintained animals on Nematode Growth Media (NGM) plates seeded with either OP50 or HB101 *Escherichia coli* and cultured plates at either 20°C or 25°C (Brenner, 1974) .

**Table 1 |** Strains used in this study.

**Table:** 

**Strain**	**Genotype**	**Source**
N2	Standard *C. elegans* wildtype strain	CGC
CFJ42	* kstSi42[ cbr-unc-119 (p1, spc2)(-)] II; unc-119 ( ed3 ) III *	our lab

**Molecular Biology**

We designed all vectors *in silico* using the molecular biology editor ApE (Davis & Jorgensen, 2022) . As criteria for selecting candidate germline promoters, we picked a mixture of trans-spliced and non-trans-spliced genes (Bernard et al., 2023) with high absolute and relative germline expression from Serizay et al. (2020). We further filtered candidates with promoter regions of 1 kb or less (defined from the start codon to the start or stop codon of the upstream protein-coding gene). Using single nucleotide substitutions, we modified homopolymers of 10 bp or more and *BsaI* restriction enzyme sites from endogenous sequences. We flanked all promoters with donor *BsaI* restriction sites and overhangs and synthesized them as gene fragments (Twist Bioscience, CA, USA). We synthesized the destination plasmid as a clonal vector (Twist Bioscience, CA, USA) and included the appropriate acceptor *BsaI* sites, a consensus start site (aaaa), a *gfp* with two nuclear localization signals (SV40 and *egl-13* ), a *tbb-2* 3’ UTR (Merritt et al., 2008) , and a universal MosTI backbone (pSEM246 **)** that contains a non-rescuing *cbr-unc-119* fragment and homology arms targeting a MosTI safe-harbor landing site on chromosome II (El Mouridi et al., 2022) . The *gfp* was codon optimized as in Fielmich et al. (2018) and designed following guidelines in Aljohani et al. (2020) but without PATC-rich introns. We generated repair templates using NEBridge® BsaI-HFv2 Golden Gate Assembly (New England Biolabs Cat. # E1601S) (Engler et al., 2009) . Final expression vectors contained an identical 15 bp stretch, which include a partial attB1 site and the consensus start site, between the endogenous promoter sequence and the start codon of *gfp* . All vectors were verified using restriction digestion and Sanger sequencing.

**Table 2 |** Candidate promoter sequences. The promoters contained 15 bp preceding the *gfp* (underlined). Substitutions to modify ≥ 10 bp homopolymers and *BsaI* sites are in bold. The consensus start site is underlined and in bold. The start codon of *gfp* is in uppercase.

**Table:** 

**Gene**	**Promoter Sequence**
*eef-1A.1*	tgtttctgttaaattaatgaatttttcataaaataaagacattatacaatataaaaatgaagaatttattgaaaataaactgccagagagaaaaagtatgcaacactcccgccgagagtgtttgaaatggtgtacggtacattttcgtgctaggagttagatgtgcaggcagcaacgagagggggagagatttttttgggccttgtgaaattaacgtgagttttctggtcatctgactaatcatgttggttttttgttggtttattttgtttttatctttgtttttatccagattaggaaatttaaattttatgaatttataatgaggtcaaacattcagtcccagcgtttttcctgttctcactgtttagtcgaatttttattttaggctttcaacaaatgttctaactgtcttatttgtgacctcactttttatatttttttaatttttaaaaatattagaagtttctaggataattttttcgacttttattctctctaccgtccgcactcttcttacttttaaattaaattgtttttttttcagttgggaaacactttgctcactccgtagcagcc aaaagcaggct **aaaa** ATG
*mex-5*	ctgcaagaaaatacattttcgactgattttacggttttcacaacggcaaaatatcagtttttaaaaaattaaaccataaaacaaataatataacccaatttttacatcaaaccacaagaaaaaaatacatttgggcccacggataaagaaattaaaaaaatacattttttaaaggcgcaccgaattaaaattcatttgggtcttaccgcgtataccgtactccgtttgtttgatcatttttgtcagcgctggcggttgttttttcatttcatttctgcttcaaagacgttttctcgaataatttttcgtttattctcttttttaaaattaatttctagccgtaaatgttataaattcacccatttaacgcaaatttcatggtaatctcatggaaaaatgcagtttctttgttaaagaaagcttaaatagcaaaaattccccgactttccccaaaatcctgctcgattttccgttttctcattgtattctctcttaattaattttatcgataatcaattgaatgtttcagacagaga aaaagcaggct **aaaa** ATG
*his-61*	gatgagaatggacttgaatcaagtatgaggaaatggtcggagcggcagatatttaagtaaaaactgcggtggatgggcggagcttcctgcagggacacatttcagagtgcacacaacactccgaatgcaccgatgcatgtaaatttgagacacagagactctgtgtcgcagacaaacaatgaggctagagataacaggggagggtgagggacagagaaccgagaggtgtccccgcagtaagctccgccccgccaccgaagaaatgaatataaacagacagtttcggttcattcttcagattcgtcttacgaacaccaaccgctcatc aaaagcaggct **aaaa** ATG
*his-64*	tgcgtttaggttgataatccgtgggggactgtaaaaaaaaagaaaccaagctgcctttgtacgcaggtgcggtgggagggcgggcgcactactgtccttgtggcaagtagaacattcggtgtgttacagacaaaggaaaggtgtcctcgcagcgtggtgcaatgtgactccgcctacccaccacagttgtgtacaaaaggaatactgtgatgtcatttcttcatctcacttgtgagtcttcaactagttcaaca aaaagcaggct **aaaa** ATG
*hil-4*	ttcttataatttgatttcgcttaagtaagagtattcctaaaaacggaaagaaaattattcaaagttgaataaagaatttttatcgcggtaatcacacttttgaaattctatgacattaaaagtcaaggcaaaattgtagatcccgtgtaatttcaattttatcaccatattaaaattcgaaattttattaaaatctgcatgaaactcatatattcagaaagtgaaaatatttatctattagcaaataagttcaggatttcaaaaacttcagatatcattgctatttaaatgacgatttgaatggtaaatgtccaactgaaaaatctctcagaactttaaatgcctgtatttatatcaattcgagagtaatttctatcgattgttggcttaatttgttgctaaactattcatctttaagattctgaaaagaaaattcaatttcataaagaaccgagaaagttatgcgtgatttagtcaagtgccctcaagtttcgcgccatttcgcattcggcgccaaacaagatgtgtcccgacagggtggtagagcgcgagtatataaggatggcgcgcgctcatttctcacactcactctccaagtgaccactactcatc aaaagcaggct **aaaa** ATG
*rla-0*	agaaattattctttcctggttttttgtctcttgtttcttatggtgtaaagtaactttatttgcgatgttcagctatttcaataaattatttgtcgttcttttatacatttttgaaagcgccacacattcaatacgtccgcacccttataaatgatcatatctaatattcccaaccaggagttacagtattcaaaaaaatttatattagaaccgagctcggcgacgagcgaaatcattctaaaaagaaaactgattaaatgtagaagtatatacaccgattttctgattttgacatcaccggactcgaatattttaattggtgttgaaattaatcaataatttctctgttttttatttagaaatatcatgatttggaggttttccctccactttcaacaaaattaatgataattctggtgtcgatttacgagaaaagttaaaggcgcatggctgctgccgccttgagggt **a** tcgtgacgaaacacattttctgttgttgagaggcgaaacattcagtgttcctaaaattttttcactgtttttccttttccttgttgaaaatgtgatcgattttttcaaaatttgattaatttatattcaaaaatgtgaatccgttcaattctgttgcactaaaaccaccgattttcaaaattgtacattttcagacctcagttttaatataatttattttttcagtctttaaaacgtaaccgcacgcgatccttaattcaccaag aaaagcaggct **aaaa** ATG
*rpl-7A*	gttttaagtcgctgaaaatctacatgtggaatatataaaaaattattaagcaaggtccaacgacaaaaatgatccaaaaagcgttttctgagttgaaaactcgaaaatgttgtttcgttttcgcgaattttaagacaaaaaactcaatttcaaacagcgcgcgcaaaaggcgcagcatgtgcgccctgccgcacacatttcgctactgactatcaaacgttgaatactcagtcaacgactgtccttggcctaaaaatcgtcgctcattttacttttgggcccgtcgtcc aaaagcaggct **aaaa** ATG
*Y37E3.8*	tctggtaaatggaagttaataacttggacaaaaaatgggaaatagaaagagaaatgaacgagctatgatgcaaaattgataataatcctaattatcacgaaattagatggatgaattaaatggtgtcaggaaatctaaattgaaaaagtgagtttagtcgagtaaaattagaaaacaaaaaatacttcaaaaataattacggaatgttataaataatgcaacacaaagtatttttcgactttctccacgaataaatcgataataacagtgagcctgaatcaaaaagtacaaaatatcaataacttatttatttgcaaaattatttaattaaattttaactaacttttctgcttttttagcttaaaaaatcgatttaaatgatgaaataagttgcgcaaaaaataaaagagtttaaagggaggctgcatttcatcgagagggt **a** tcgccacgaaatgcgcatcaaacgagtgtgaccgtacctcagattttgggagacacactcactgaatttttgcgaatattttaggatttctttttgttctcaatggtttttcgctaggttttaatgaaaaaagtgtgtgatctggtcgattttaattatctttaaagacaaaatttgtatttttgtagtaattttatccgagaatatcgggaacctttcattgaatatttttctctgatttttcttctccattttcgatcgaaatttgccgcttttacacattcaaattgtttactttacagggt aaaagcaggct **aaaa** ATG
*klp-19*	tgaaaattattttaaaatcaatatatttagtgataatttgctcattttcgaaggtttatggcttaatctcgggaagaaaacatgttaaaatacagagaaaagtgagcaaaatgatgaagaacttcttcattaattaaataaaaacagaaaaacattttt **g** ttttcactgggtccgcaattgtgcgtgaactctgacgtcttgtccgtaaatacactcgcggcgcgaaattttgatttcaaaagcctccgccccc **a** ccccctctcattcattcgtgaaaaatcattcattttcgtgttttcttcccgag aaaagcaggct **aaaa** ATG
*his-68*	tgttgagtgattggctgaagactcacgaatgatgatgaactgggaaaacagttcttcttatatactccctatgcagatacagcggtgcaaaggcggagtcagtttgcgccatcctgcagggacacatttccgtgcctgccgctcttgcatctgcagagatatctgccgttttggcctctagtaggcgagacatacaggaaggtgagagagacgcagaaacgagataaatgtgtccccgcaggaatttaccgaatgttcaaaaagaggtgtccccgcaggaaactccgccccgccaccgtaaaaataaatataaagcgactgtcttggttcattcttcagattagttttacgatcaccaatcgctcata aaaagcaggct **aaaa** ATG
*W05F2.3*	tctgcaatcaaaaatatcgattttaatctgaaaactggacggaaaataggggaaaaatttcgaaaataaataggaaaaaattgttttcaaataaacaacaactacggtagcacattgcggagtggcggtgtttgcgtactttttcaaatttgcgcgcgaaaatttgagttttttcgccattttttgcgggaatttttggattttcttttgttttgctcgcttttttcagtttttcaaagtcatttcttcatttttcttcaaatttacctctcctctatttaaaaaaagttaaaattttcag aaaagcaggct **aaaa** ATG
*spn-4*	tctttataatattatcatttcttttgcaaagtatctgaaagaaactataagaatgagaaaaaatagataaaattttatgggaaaatgggaaaagaattcaataaaaaataaatgcagagaaaaaaaacgcaattgacatgaaacgagaaaaagagaagaaaggcaactgcgtgcacgctccttgcaaaaactgcgtgccaggattgcgaaggcggggcttatgaatgaagcgcgagtttgaatcaaataaaattattttttgctcttggcctaaagaaaatcgaattaaagctttagtttctagtaattcgtaaaataatttttttaatcgaaaatagaacacaaaattgtctagttatattaacttttagacctgatccgacgttcgtatgaactagaaatctagaagatcacaaaggcgcgaaattttgattttagatttttctgtaactcggtggcgggaaaatgtgaaattatattttcaatcgttttatcatcatttggaatcatttctgcacttgtcttctgttgtcttgtcttttctttacccagttcctcatttttctttattcaactttttttttcaga aaaagcaggct **aaaa** ATG
*pos-1*	tttgaaactgaatcaaagaaaattaacttttaaaatgaggacaaacacagaacgtggaaaatgtagaaatggaaaatcacggtggtacagcagctagatgagtgcaaatgcgctctaccgaacaaacccaaatttgaccggttaggaaattttcaatcaaaaaaattttcaatagaaaagatagagaattttcttgcggccatttttcatatgcatcgcaaattcaaatttggggttagttttcgagatagcagccaatataggttttcagactgagtatcataaaccaagccatctagtttctgtgctaccatgaaatccaacacggcgatgcgaacacgctctattgcgaaaattcaagtttt **g** tttttcaaaatttctcaaaatttgatcatgatttcgagcggtattttttaatttttactttttagtttccgcgaatttttgataagtaaattttttcgctcactctttgttcatttcattcagatgttccatttcaaggatttttttcctttttattttcttttctgccggtgctcccaagttttcttgatttatattctcttctttttccatcatcatcacagtttgtaaatttatttttctagattcaaa aaaagcaggct **aaaa** ATG
*Y75B12B.1*	attttttgttcggaatcccccaatggaaaactttaaattattctaaaaggccttaaaaactggtaaataacaaataaatgaaatatttattttttcttcaggaaaaatgatgaaaaacaagctggaaggcgtggcaaaaatccggaagaaaacatgttttgcatattcgcgtcaaatagagtgttcagtctgcagttgcacatgttcttgtctacagtagtatgagagcgcgtgtggctaatccgcccatttttgcaagcgccgcacgctttttctgcaaccacgcgtcacgtggcacggttcaaacatagtttttcgaaactcgaaccttgcaaatactttttcttttcctagaaatgtgtttcttttgttagatttt **g** tttttcaaaacatcggaaaacggaaaatttttgttgttcggtttaaaaaatcagaacttttt **g** ttttcagaaaagaaatttgatcagatctatataattttgacctatccgtagatcaaataagtttattctgcgcgcggtaaaaattgaattctttttcgtttcttttctcctttgtcccaaatttggtccagctctaactttttttctctttccctggttttcccatgtttcccttccattttgcactatgtattacgcttttcattaattttcattttcagatt aaaagcaggct **aaaa** ATG
*puf-5*	gaatagttctgatcagtcagccatttctatttttctgtggccccttcatgttgtaaataaatgtattatttttttgggaaaacttcatttattcgggcggtttcactgaaaactgtttaatgtctcttcagacagaaacacaatttcgacgagaaaagtgcagtcttatcttactgtagctttaaaggcgcacaccaatattttactcaatcgtagttcgcgctgtgagaacatttggaccaaacaagggattttttgcaaaaatcattccatcaaacgcttcatttcgtattattgctgcttaaaaacaatttgtatgacttaaaattggctttaataatgttttttttcaataatcttaattttcagacataaatatcaattaaaactttgatctactctgtttgatctgccgattttgatcaccggcgggaatattttgcatttaattttctattcgccggctgctctcatttcataatttcatttcattcagtatctccttttttcctaaaatattgtgttttatcgaagttgtatttatttctttagatactaacctgtactcctgtgactctgt aaaagcaggct **aaaa** ATG
*mei-2*	agaactaaattaaatattaaatatatcgtttcaagaattcattggaatgaggcaaaagtaaatacttaggattaaaaaatccagctttatattaaaaactttaaaggcgcatatgagatgttattcgggtcccgcagcgctcatgcggggtacgatagtacttcaaagaattacgcgggaatttcttttatgcgggaaaacggttttttcttgtttactagttcctttctttcgtctaattttgatatcttgtgtttttttccaattataaaatgtttgtctcttcttaaatttgaaattttgaaa aaaagcaggct **aaaa** ATG
*mesp-1*	cttaatttgagttttaagcaaagaaacagcgctgaaaaattcaaaaataatatggccgggaaaattgtttagggtaaaaatcgagttttctcggccacggccacgaagtattaacgcgctaatttcaaattttgaagtatcatttaatcacgcccagtctcgtggtggacccgtccggaaaaatcaacgtgcctttaatctcgcgcatctgagtctctactttttacatgttctctcacttgtgaagagtacggtatcacgatttctagacgctctaggttttatttttataattttgatctaatattaatgcgcactgatctgaatttcgccgccaattattttaacatgatttttccttgttgacaattcatattcatttcattttttcacatctacgagctttttcttattattttaatttttatcgttctctcttaatttcctctctatcttttatttatcaaaatcgaatatttcttgcagatgcttgcgattcccaaaactccaaccaggaac aaaagcaggct **aaaa** ATG
*puf-11*	tttttctcgttgtgttcattagtttacagctttatcctgtaacatggcatgttctatatcatcatgtacaatgttttattgtctagagttttattttcctttttgctccattttgtatccttggttttctttttaaaattacagtcattcgtaaataaattgactggtgttgttagaatttttcccacgtcccagtttcaggatacttgaatgtattccatagtttccatcaattggccgatctaattgtatgaaggattcgtattcttttgctgagtcccgctccgaatatcttttggcatttaaatctaaaaattttctgcaataagcaccaggaaccggacatgcttcatgagctcataatcataactactagcttctgctaattctgcagtcaacccgagatcgtaaccataagtcgtggaactgagttcttgaagtttgtagatgcccgcgccactgattcgggtcatgaatccgcgttgcagttgaaaatattctattattatatttaattttgttctttgatttaacaaaaaaatagcgctcaaatacgcttaaaacaagaatttttcgtattctgtgccaaaatttatcaatcgtggcgag **t** cctataggcttaacgtcgaaatgcgccttaaaattctacagtactttatttcattttgcgcgggaaattttgggattttagttttaaaaacgcgcgaatttttgcattttggttcctgatttcttcatttctgctcattttttgccttcgaggacgtttcccaaaattttgctctcattttcattaattctatcgtattttccacgtattccgtccattcaaacgaccatttttcccaaaaaaatattttcagttagtctccgtaccaat aaaagcaggct **aaaa** ATG
*F23A7.8*	ctccgctcccaaaaaa **g** aaaaaatttgcgttttcgcgctttttcgttttcctcaaaaacacgacatcggtgcattttttaattctgcattttttaattttgaaggtcacgagttcaagtccggcctagtccccccaggtttgctcagcctggagattgttgagtattcattgggtcttccatcaaacccaatgtcttatgtcgatggacgaaacaaaaatcgaaagggctattctagagttggagcttaactccactcccaaaaaaaaattgttttttacgctttcgcgcgttgttgttattcttgaaaactcggcatttacgtttctctcgaaagtaaaatttcagtgcactctttaattccgatatctgaaaaccaacatttaataatacggagatatatatcccacaaatgttcaaaactacagtactaccgtactcctacagtacccctatagtctcactgcagtaccccgacactactcaacattctgagacgtacgagcttgccgcttgcatttatcgttttccgcgagcggttatgtaaattccaattaagtc aaaagcaggct **aaaa** ATG
*clec-87*	gaaaattgcaagggaggagagaattgaattcgaatctcgagcgttattttctgttgaaaatgggcgcattaagtgcgctccactgcatattttgttaatatcaagggcggctattcaaaatataattttcaaaattaattacaattttttaaaacaagatcttgcccttttaaaagttaaatattttcataaatgaaaatctgcagtgtgctacagaacatttaaaccatcatccgtgcttcaaattcataacaccagttcagaaatgaagtttaggatatttctagacttgatttgttttgaaattattatttgatctctgtctattagcggcgcgcatttgtgaaaatttcaggaaacggcggcaaaaatgtgcataaaaggagaacgaaacgttcatttcctcagtcttcac aaaagcaggct **aaaa** ATG

**Software**

An online application for designing transgenes is available at www.wormbuilder.org/transgenebuilder. A manuscript detailing the application is in preparation by the authors (Vargas-Velazquez, El Mouridi, Alkhaldi, and Frøkjær-Jensen). The version described in this manuscript has been archived in Caltech Data - 10.22002/qs7eh-g0669.

**Single-Copy Insertions**

We generated single-copy insertion of candidate germline reporter transgenes using MosTI. The technique relies on the reconstitution of two non-rescuing *cbr-unc-119* fragments: one located in the genome in a safe-harbor landing site while the other fragment is co-inserted with a transgene of choice using CRISPR-Cas9. Following a double-strand break and homology-directed repair, successful single-copy insertions events are marked by the phenotypic rescue of the *unc-119* gene (El Mouridi et al., 2022) . In brief, for each candidate, an injection mix contained 25 ng/μl pCFJ2474 ( *smu-2 p::Cas9:: gpd-2 ::tagRFP-T* ), 10 ng/μl pSEM235 ( *mlc- 1 p::mCherry* ), 10 ng/μl pSEM238 ( *snt-1 p:: HisCl1* ), 15 ng/μl pSEM318 (MosTI sgRNA), 10 ng/μl repair template, and 30 ng/μl 1 Kb Plus DNA Ladder (ThermoFisher Cat. # 10787026) for a final DNA concentration of 100 ng/μl (Mello et al., 1991) . We injected each mix into 10-20 young adult CFJ42 ( *kstSi42[ cbr-unc-119 (p1, spc2)(-)] II; unc-119 ( ed3 ) III* ) animals that were maintained at 20°C on HB101 . After injection, we placed single animals on NGM plates seeded with OP50 at 25°C until starvation. We added 500 μl of 50 mM histamine dihydrochloride (GoldBio Cat. # H-110-100) to plates with *unc-119* rescued animals. After 1 hour, we screened plates for sensitivity to the negative histamine selection marker (El Mouridi et al., 2021) . We singled *unc-119* rescued animals lacking pan-muscular, *mCherry* , co-marker expression (El Mouridi et al., 2020) and insensitive to histamine onto fresh OP50 plates and propagated insertion lines until homozygous. We generated one to four independent insertions for each transgene (exact numbers are in parenthesis) and detected no observable differences across independent lines: *eef-1A.1 p* (1), *mex-5 p* (3), *his-61 p* (2), *his-64 p* (2), *hil-4 p* (3), *rla-0 p* (3), *rpl-7A p* (1), *Y37E3.8 p* (1), *klp-19 p* (1), *his-68 p* (1), *W05F2.3 p* (3), *spn-4 p* (3), *pos-1 p* (4), *Y75B12B.1 p* (1), *puf-5 p* (4), *mei-2 p* (1), *mesp-1 p* (3), *puf-11 p* (2), *F23A7.8 p* (3), *clec-87 p* (3).

**Fluorescence Quantification**

We selected one independent insertion line for each candidate promoter for fluorescence quantification. We noted early germline GFP expression using the *puf-11* promoter that was rapidly silenced. We, therefore, grew the strains for multiple generations to ensure stable transgene expression. In order to minimize bias during quantification, we blinded N2 and transgenic insertion lines to their genotype. We then synchronized them by egg prep in a drop of bleaching solution (Stiernagle, 2006) . The following day, we moved five animals to 10 fresh OP50 plates per strain and cultured them at 25°C for five days. *Visual Quantification* : We scored three plates per strain with a mixed-stage population by eye on a Kramer Scientific FBS10 LX microscope equipped with objectives 2x (Plan APO Objective 0.055NA), 10x (Plan APO Objective 0.3NA), and 20x (Plan APO Objective 0.42NA) and an X-Cite 200DC illuminator using the following scale: 0 (no detectable expression at 20x), 1 (detectable at 10x with zoom), 2 (detectable at 10x without zoom), 3 (detectable at 2x with zoom), and 4 (detectable at 2x without zoom). *Flow Cytometry* : We washed seven plates per strain with M9 to remove particles and bacteria twice. We measured the fluorescence of a mixed-stage population using a COPAS FP-250 μm large-particle flow cytometer (Union Biometrica) equipped with 488 nm and 561 nm excitation lasers. To select adult worms, we filtered raw data by TOF (time of flight) between 1500 and 1800 and peak-height extinction below 35,000 using Microsoft Excel for Mac (v16.70). We subtracted the mean peak-height GFP measurement from non-transgenic N2 animals from all reported values. We generated plots using GraphPad Prism 9 for macOS (v9.5.1).

**Imaging**

We immobilized animals using a 50 mM sodium-azide in M9 solution and mounted them on 2% agarose pads. We took images on an upright, non-motorized, compound microscope (Leica DM2500 with a Leica DFC7000 GT camera and Leica SFL4000 LED light source) with a 20x air objective. We maintained constant exposure time, gain, and binning in all images.

## Reagents

**Table 3 |** Plasmids generated in this study (sequences available on request).

**Table:** 

**Plasmid**	**Transgene**	**Description**
pMDJ303	* nls::ce-gfp::nls:: tbb-2 3’ UTR *	*BsaI* destination vector (Amp ^R^ )
pMDJ304	*mex-5 p::nls::ce-gfp::nls:: tbb-2 3’ UTR *	Candidate germline promoter repair templates in a universal pSEM246 MosTI backbone (Amp ^R^ )
pMDJ305	*eef-1A.1 p::nls::ce-gfp::nls:: tbb-2 3’ UTR *	
pMDJ311	*spn-4 p::nls::ce-gfp::nls:: tbb-2 3’ UTR *	
pMDJ312	*his-68 p::nls::ce-gfp::nls:: tbb-2 3’ UTR *	
pMDJ313	*Y75B12B.1 p::nls::ce-gfp::nls:: tbb-2 3’ UTR *	
pMDJ314	*F23A7.8 p::nls::ce-gfp::nls:: tbb-2 3’ UTR *	
pMDJ315	*mei-2 p::nls::ce-gfp::nls:: tbb-2 3’ UTR *	
pMDJ317	*puf-11 p::nls::ce-gfp::nls:: tbb-2 3’ UTR *	
pMDJ318	*puf-5 p::nls::ce-gfp::nls:: tbb-2 3’ UTR *	
pMDJ319	*mesp-1 p::nls::ce-gfp::nls:: tbb-2 3’ UTR *	
pMDJ320	*klp-19 p::nls::ce-gfp::nls:: tbb-2 3’ UTR *	
pMDJ321	*W05F2.3 p::nls::ce-gfp::nls:: tbb-2 3’ UTR *	
pMDJ322	*his-61 p::nls::ce-gfp::nls:: tbb-2 3’ UTR *	
pMDJ323	*clec-87 p::nls::ce-gfp::nls:: tbb-2 3’ UTR *	
pMDJ324	*his-64 p::nls::ce-gfp::nls:: tbb-2 3’ UTR *	
pMDJ325	*Y37E3.8 p::nls::ce-gfp::nls:: tbb-2 3’ UTR *	
pMDJ326	*rla-0 p::nls::ce-gfp::nls:: tbb-2 3’ UTR *	
pMDJ327	*rpl-7A p::nls::ce-gfp::nls:: tbb-2 3’ UTR *	
pMDJ328	*pos-1 p::nls::ce-gfp::nls:: tbb-2 3’ UTR *	
pMDJ329	*hil-4 p::nls::ce-gfp::nls:: tbb-2 3’ UTR *	
